# Developmental timing of extreme temperature events (heat waves) disrupts host–parasitoid interactions

**DOI:** 10.1002/ece3.8618

**Published:** 2022-03-18

**Authors:** Megan Elizabeth Moore, Christina A. Hill, Joel G. Kingsolver

**Affiliations:** ^1^ University of North Carolina Chapel Hill North Carolina USA

**Keywords:** climate change, developmental timing, extreme temperature event, host–parasitoid interaction, parasitoid

## Abstract

1. When thermal tolerances differ between interacting species, extreme temperature events (heat waves) will alter the ecological outcomes. The parasitoid wasp *Cotesia congregata* suffers high mortality when reared throughout development at temperatures that are nonstressful for its host, *Manduca sexta*. However, the effects of short‐term heat stress during parasitoid development are unknown in this host–parasitoid system.
2. Here, we investigate how duration of exposure, daily maximum temperature, and the developmental timing of heat waves impact the performance of *C*. *congregata* and its host¸ *M*. *sexta*. We find that the developmental timing of short‐term heat waves strongly determines parasitoid and host outcomes.
3. Heat waves during parasitoid embryonic development resulted in complete wasp mortality and the production of giant, long‐lived hosts. Heat waves during the 1st‐instar had little effect on wasp success, whereas heat waves during the parasitoid's nutritionally and hormonally critical 2nd instar greatly reduced wasp emergence and eclosion. The temperature and duration of heat waves experienced early in development determined what proportion of hosts had complete parasitoid mortality and abnormal phenotypes.
4. Our results suggest that the timing of extreme temperature events will be crucial to determining the ecological impacts on this host–parasitoid system. Discrepancies in thermal tolerance between interacting species and across development will have important ramifications on ecosystem responses to climate change.

## Graphical abstract

Extreme temperature events can have important effects on species interactions, especially when species differ in thermal tolerance. Thermal tolerance is not static across an organism's life time, so that some developmental stages are more sensitive to temperature than others. We investigate the effects of heat waves on a host–parasitoid interaction and find that the parasitoid has a lower thermal tolerance than its host, but only at specific developmental stages; this indicates that the timing of heat waves is crucial in determining the outcome of the host–parasitoid interaction.

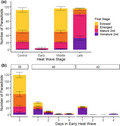

## 1 INTRODUCTION

Extreme temperature events are increasing in frequency (Fischer & Knutti, 2015) and have impacts across ecological scales (Bailey & van de Pol, 2016; Hoffman & Srgo, 2011; Kingsolver & Buckley, 2017). During these events, daily maximum temperature (DMT) can exceed the upper thermal limit of organisms in that environment. Even short‐term exposure of a few hours to high temperatures can prove detrimental to ectothermic organisms, such as insects (Colinet et al., 2015; Ma et al., 2015; Roux et al., 2010). Thermal sensitivity and thermal tolerance are not static over an organism's life span; different developmental stages can have different responses to the same thermal stress (Bowler & Terblanche, 2008; Kingsolver & Buckley, 2020). The timing of heat wave events, therefore, can lead to drastically different outcomes, depending on when during ontogeny the stress was experienced. Recent studies in insects have shown that heat shocks experienced at different life stages can have differing phenotypic and fitness effects, with no clear pattern across taxa (Banahene et al., 2018; Klockmann et al., 2017; Knapp & Nedvӗd, 2013; MacLean et al., 2016; Moghadam et al., 2019). As global temperatures continue to rise, and extreme climatic events increase in frequency, understanding the responses of organisms to acute versus chronic thermal stress will be imperative for creating a predictive framework around climate change (Ummenhofer & Meehl, 2017).

Beyond the impacts on individual organisms, climate change will affect and potentially disrupt ecological interaction between species. Parasitoid insects are vital top‐down regulators of many insect herbivores, and there is a growing body of evidence that they have greater thermal sensitivity and lower thermal tolerance than their hosts (Furlong & Zalucki, 2017; Jeffs & Lewis, 2013; Mutamiswa et al., 2018). Parasitoids deposit their eggs on or in a host organism (usually another insect or arthropod), which they use as a food source during development, and inevitably kill (Godfray, 1994). Because parasitoids rely on complex physiological mechanisms to survive within their host, they are especially vulnerable to increasing temperatures, unpredictably variable temperatures, and stressful temperature events that could disrupt these processes (Le Lann et al., 2021). Many parasitoids also rely on endosymbiotic viruses to manipulate elements of host physiology and behavior; if high temperatures have negative effects on these viruses, the outcome of host–parasitoid interactions will be altered (Seehausen et al., 2017).

The question of how climate change will impact insect parasitoids can and has been approached from many angles: parasitoid phenology (Jeffs & Lewis, 2013; Wetherington et al., 2017), parasitism success and survival (Delava et al., 2016; Iltis et al., 2018; Moore, et al., 2021a, 2021b), resource use and behavior, (Jerbi‐Elayed, 2015; Le Lann et al., 2014; Moiroux et al., 2016; Valls et al., 2020), intergenerational effects (Iltis et al., 2020), and chronic exposure to heat stress throughout development (Moore et al., 2020, 2021a, 2021b; Seehausen et al., 2017). In our study, we specifically investigate how heat waves (defined here as short‐term heat events where the daily maximum temperature exceeds the thermal optimum) impact the survival and performance of a parasitoid wasp at different stages of larval development within its host caterpillar. The specific effects of high temperature stress across parasitoid life stages are not well understood and are only beginning to be explored, but will play a critical role in how climate change affects parasitoid populations (Zhang et al., 2019). Due to the life history of parasitoids, their performance is inexorably dependent on their host, and for many, on their viral endosymbionts. Each of these ecological players has a baseline sensitivity to temperature, which is altered by the timing and nature of the temperature stress. The question of how these complex, shifting thermal sensitivities affect the ecological relationships between parasitoid, endosymbiont, and host in the face of heat waves remains to be fully explored. Here, we focus on the organismal responses of the parasitoid and host, but have structured our experimental designs with the timing and function of viral action in mind; direct tests of the effect of temperature on the viral endosymbiont are being investigated, but are outside the scope of this study (Malinski et al., 2021).

We aim to investigate this question using the model host–parasitoid system of the larval tobacco hornworm moth (*Manduca sexta*) and the braconid wasp *Cotesia congregata*. The physiological processes of parasitism have been well studied in this system, and the thermal biology of the host caterpillar is well understood (Adamo et al., 2016; Beckage & Riddiford, 1978, 1982; Beckage et al., 1994; Dushay & Beckage, 1993; Kingsolver et al., 2015, 2016; Kingsolver & Woods, 1997; Potter et al., 2011). The parasitoid wasp relies on an endosymbiotic polydnavirus (CcBV) to control key aspects of *M*. *sexta* caterpillar development, physiology, and behavior, especially early in parasitoid development when the virus must suppress the host immune system for the parasitoid eggs to survive. *C*. *congregata* larvae feed nondestructively on nutrients in the host caterpillar's hemolymph and eventually emerge through the host cuticle to spin cocoons and pupate (Alleyne et al., 1997; Beckage & Riddiford, 1983). Stressful temperature environments are likely to be detrimental for the parasitoid wasp at various points in development, due to disruption of viral action, or via stress on the parasitoid larvae themselves. Recent studies have shown that *C*. *congregata* has lower thermal tolerance during rearing than its host and that exposure to high, fluctuating temperatures throughout development results in complete wasp mortality and abnormal host phenotypes, most likely due to disruption of vital polydnavirus functions (Malinski et al., 2021; Moore et al., 2020, 2021a, 2021b).

Here, we investigate the effects of short‐term, high temperature events (heat waves) where the DMT exceeds the parasitoid's thermal optimum. We examined the effects of different DMTs, the duration of exposure, and the developmental timing of heat wave. For our study, we identified two critical points in development that are likely to be temperature‐sensitive: (a) directly after parasitism when the parasitoid wasps are eggs, and the polydnavirus must suppress the caterpillar's immune function, and (b) during the parasitoid wasps’ 2nd larval instar (prior to emergence from the host), when nutrient uptake and host hormonal manipulation is crucial for successful emergence from the host (Beckage & Templeton, 1986; Bentz & Barbosa, 1992; Dushay & Beckage, 1993). Our studies test three hypotheses: (a) Parasitoid sensitivity to heat waves will differ across ontogeny: high heat stress will be most detrimental early (during embryonic development before hatching/viral immune suppression) and late (during larval nutrient uptake before emergence from host) in development. (b) Exposure to high DMT early in development will cause wasp mortality prior to hatching and cause abnormal host phenotypes (Moore et al., 2021a, 2021b). (c) Increasing the duration of exposure (multiple days in heat wave) will increase the frequency of wasp mortality and abnormal host phenotypes.

## 2 METHODS AND MATERIALS

### 2.1 Study system

*Manduca sexta* (Lepidoptera: Sphingidae) hosts were obtained from the University of North Carolina—Chapel Hill laboratory colony which has been maintained under laboratory conditions at the University since the 1980s (>250 generations) with no reintroduction of wild individuals. All life stages of the UNC‐CH colony were maintained at a constant 25°C and a 14L/10D hour light cycle. Caterpillars were given an artificial, wheat germ‐based diet (modified from Kingsolver & Woods, 1998), and adult moths were fed a 10% honey water solution.

*Cotesia congregata* (Hymenoptera: Braconidae) is a gregarious endoparasitoid of *M*. *sexta* and several other Sphingid species. Female wasps oviposit multiple wasp larvae (50–200) into the hemocoel, which feed nondestructively on host hemolymph (Alleyne et al., 1997; Beckage & Riddiford, 1978, 1983). Before emergence from the host cuticle, wasp larvae manipulate the host caterpillar to cause cessation of feeding, locomotion, and development. The caterpillar remains alive throughout wasp pupation, but inevitably starves to death. *Cotesia congregata* wasps were obtained from the UNC‐CH laboratory colony maintained since 2017 (Moore et al., 2020, 2021a, 2021b). *M*. *sexta* from the UNC‐CH colony were parasitized at the 4^th^ instar to perpetuate the parasitoid colony. Adult wasps, cocoons, and nonexperimental hosts were maintained at room temperature (~25–26°C) and 14L:10D light conditions.

### 2.2 Experiments

**FIGURE 1:**
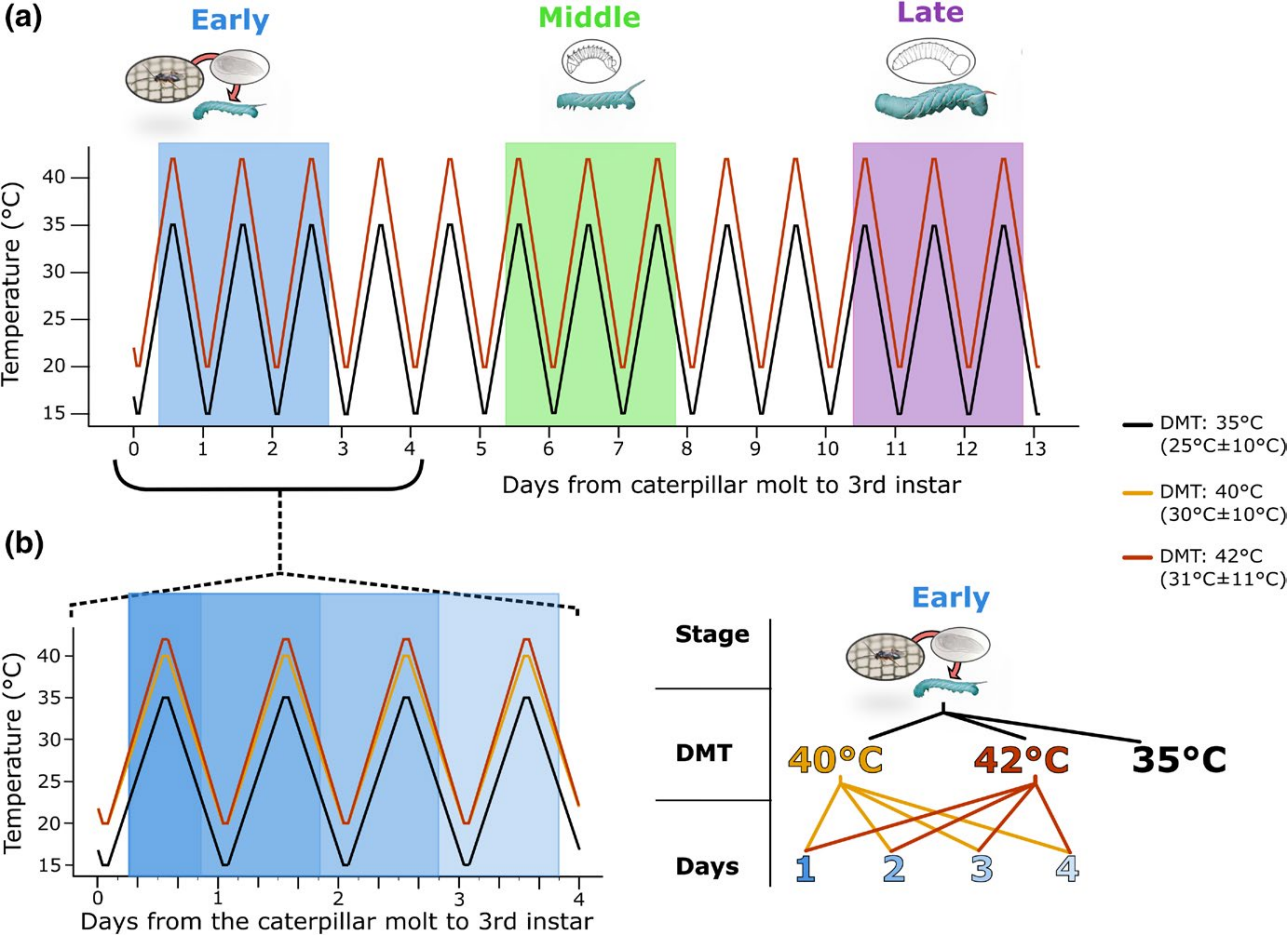
Experimental design and set up for the developmental timing experiment (a) and temperature and duration experiment (b). a: For the developmental timing experiment, caterpillars were parasitized by *C*. *congregata* wasps on the day they molted to the 3rd instar. Parasitized caterpillars were exposed to a 3‐day heat wave with a DMT of 42°C (31 ± 11°C), at different stages of development: early (same day as oviposition, while the parasitoid wasps are eggs), middle (5 days after oviposition, wasp larvae are 1st instars), and late (day 1 of the caterpillar's 5th instar, wasp larvae are 2nd instars). After the heat wave treatments, experimental caterpillars were returned to the rearing treatment (DMT = 35°C, 25 ± 10°C); control caterpillars remained at the rearing treatment throughout development. b: For the temperature/duration experiment, caterpillars were parasitized by *C*. *congregata* wasps on the day they molted to the 3rd instar. Parasitized caterpillars were exposed to one of two heat wave regimes on the same day as oviposition: DMT = 40°C (30 ± 10°C) of DMT = 42°C (31 ± 11°C) for 1–4 days. After the heat wave treatments, caterpillars were returned to the rearing treatment (DMT = 35°C, 25 ± 10°C); control caterpillars remained at the rearing treatment throughout development

The current study consisted of two experiments: one determining how parasitoid sensitivity to heat waves varies across ontogeny (developmental timing experiment), and the other investigating the effects of heat wave temperature and number of heat wave exposures on *C*. *congregata* survival and development early in parasitization (temperature/duration experiment). The rearing and control treatment was the same for both experiments (25 ± 10°C, DMT = 35°C) and has been determined to be nonstressful for both parasitoid and host (Moore et al., 2021a, 2021b). All temperature treatments and controls followed the same thermal regime: 2 h at the daily minimum temperature from 01:00–03:00, then continual ramping to the daily maximum temperature from 13:00–15:00 (Figure 1). The ramping rates differed between the rearing and DMT 40°C treatments (±10°C) and the DMT 42°C treatment (±11°C), and were 1.82°C/hour and 2°C/hour, respectively. The experimental temperature regimes were chosen based on extreme recorded field temperatures in Chapel Hill, NC. The temperature/duration experiment was conducted in January–April, 2018, and the developmental timing experiment took place in October–December, 2018. Organisms were housed in climate control chambers (Percival Scientific 36VL) under 14L/10D hour light cycle. An open container of water was placed in each chamber to prevent desiccation of organisms or artificial diet (Moore et al., 2020). Newly hatched caterpillars were reared on an artificial diet in communal petri dishes until the molt to the caterpillar's 3rd instar. On the day of the molt to 3rd instar (day 0), caterpillars were assigned a unique ID, allocated to a heat wave treatment, weighed, parasitized, and housed individually in small petri dishes. Caterpillars were parasitized by exposing individual caterpillars to a colony of adult wasps, and observing until an oviposition event of >2–3 s occurred.

### 2.3 Developmental timing heat wave experiment

The development timing experiment consisted of three separate heat wave treatments and one control (see above). The heat wave regime was the same for all treatments: three days at 31 ± 11°C (daily maximum of 42°C for 2 h) (Figure 1a). Recent work has shown that a single exposure to this heat wave regime does not reduce survival, development time, or pupal mass of unparasitized *M*. *sexta* regardless of developmental stage (Kingsolver et al., 2021). The treatments differed in the developmental stage of the parasitoid larvae at which they experienced the heat wave temperatures (recall that caterpillars in all treatments were parasitized). Early Heat Wave started on day 0 of the 3rd instar of the caterpillar, the same day as oviposition; the Middle Heat Wave treatment started 5 days after oviposition (when the majority of *C*. *congregata* eggs have hatched); the Late Heat Wave started on day 1 of the host's 5th instar (when the majority of *C*. *congregata* larvae have molted to the 2nd instar), which ranged from 8 to 12 days after oviposition (Figure 1a). Sample sizes for each treatment ranged from 30 to 60 parasitized *M*. *sexta* caterpillars (Table S1A). Individuals in heat wave treatments were transferred from the rearing temperature (25°C±10°C) to the heat wave chamber at least 2 h before the DMT on the first day of the treatment. Caterpillars were removed at 4–5 p.m. on the last day of the heat wave treatment and were returned to the rearing temperature. Parasitized hosts in the control treatment remained in the rearing temperature (25 ± 10°C, DMT = 35°C) throughout development (Figure 1a).

### 2.4 Temperature and duration of early heat wave experiment

The temperature/duration experiment consisted of a 2 × 4 factorial design plus one control treatment for a total of 9 treatment combinations. All *M*. *sexta* caterpillars were parasitized for this experiment. Parasitized *M*. *sexta* were exposed to one of two fluctuating heat wave temperatures, one with a daily maximum temperature of 40°C (30 ± 10°C) and the other with a DMT of 42°C (31 ± 11°C). Both temperature treatments ramped continuously between 2‐h periods at the high and low temperatures in a 24‐h cycle (Figure 1b). Parasitized *M*. *sexta* were placed in a heat wave treatment on the same day as oviposition, at least 2 h before the daily maximum temperature. Experimental insects remained in the heat wave treatments for 1, 2, 3, or 4 days (i.e., they experienced the DMT 1–4 times) (Figure 1b). Caterpillars were removed from the heat wave treatment at 4 p.m.–5 p.m. on the day of their last heat wave and then returned to the 25 ± 10°C (DMT = 35°C) rearing treatment. Parasitized hosts in the control treatment remained in the rearing temperature throughout development (Figure 1b). Sample sizes for each treatment ranged from 20 to 46 individual parasitized caterpillars (Table S1B).

### 2.5 Monitoring and measurements

For both experiments, caterpillars in all treatments were provided diet ad libitum and were monitored daily for diet quality, molting, wasp emergence, or death. Artificial diet was replaced as needed. Date and mass at each caterpillar larval molt were recorded. Wasp survival to pupation and host mass were recorded 48 h after the start of wasp emergence to allow for full emergence and hardening of cocoons. At this point, wasp cocoons were removed from the host and returned to the rearing temperature until adult eclosion. Host caterpillars were frozen for dissection to determine the number of parasitoids that hatched but did not emerge (load, see below). Wasps were frozen 24 h after eclosion and the number that successfully eclosed (temperature/duration and developmental timing experiments) and the sex (developmental timing) were determined. The mass of adult wasps (developmental timing) was determined by weighing all wasps for each host (separated by sex) and dividing by the number of wasps weighed. The sex and mass of adult wasps were not measured in the temperature/duration experiment due to time constraints.

Normal parasitization disrupts caterpillar development, preventing hosts from entering the prepupal or “wandering” stage. Parasitization that is disrupted by high temperature stress can result in hosts that fail to have wasp emergence; these hosts often die as caterpillars, but some individuals show delayed behavioral and physiological signs of wandering, though all laboratory reared individuals die as wanderers or larval–pupal intermediates (Moore et al., 2021a, 2021b). Hosts without wasp emergence (WOWE) were defined as parasitized caterpillars that failed to exhibit wasp emergence a week after the caterpillar molted to the 5th (final) instar (the normal time frame of wasp emergence at 25 ± 10°C is 3–5 days after the host molts to the 5th instar). WOWE hosts were maintained in the rearing temperature, monitored daily for food, signs of wandering (cessation of feeding, clearing of dorsal cuticle), or signs of illness (discoloration, flaccid cuticle) and weighed weekly after the caterpillar molted to the 5th instar. WOWE hosts were culled and weighed 3 weeks after molt to the 5th instar, or at signs of illness. Those that exhibited signs of wandering were placed in pupal boxes and monitored daily for pupation or death. A subset of WOWE hosts from each treatment was frozen for dissection to determine the fate of the wasp larvae within the host. These hosts fell into two categories: WOWE hosts with no visible wasp larvae (indicating no parasitoid survived hatching), or WOWE hosts with visible wasp larvae (indicating that parasitoids successfully hatched, but died before emerging from the host). A small subset of parasitized caterpillars wandered, but was not defined as WOWEs, as they wandered within the normal time frame of *M*. *sexta* development (3–5 days after caterpillar molt to 5th instar). Since the prevalence of these did not differ among treatments in either experiment (Table S1), these were presumed to be the result of failed ovipositions and excluded from the analyses.

Hosts with emergence were dissected to determine the total number of parasitoids that developed within the host. Wasp larval stage was determined visually, using the features described by Fulton (1940). Here, we define the parasitoid load as the number of 2nd instar wasp larvae found within the host and are therefore underestimating the true parasitoid load. Many hosts also contained 1^st^ instar wasp larvae in their hemocoel; however, due to small size and transparency, accurate counts of 1st instar *C*. *congregata* larvae are difficult to acquire. We could not determine total load (the number of eggs deposited by the female parasitoid) in most cases, as hosts exposed to heat waves early in development exhibited low numbers of wasp larvae in their hemocoel, as well as abundant melanized plaques—an indication that many parasitoid eggs did not hatch and were encapsulated. Hosts with parasitoid load >300 were assumed to have been multiply parasitized and were excluded from analyses (*n* = 3); normal parasitoid load size ranges from ~50–200 (M. E. Moore personal observation). In the Late heat wave of the developmental timing experiment, a chamber miscalibration caused the DMT <42°C; caterpillars in this treatment that experienced the heat wave after the miscalibration were also excluded from analyses (*n* = 35).

### 2.6 Statistical analyses

#### 2.6.1 Developmental timing experiment

Parasitoid survival to emergence and eclosion were analyzed using generalized linear mixed effects models with binomial distributions using the “glmer” function in the lme4 package in R (v. 4.0.2). The number of successes (number emerged/eclosed) and the number of failures (number unemerged/load) were used as the response variable, and heat wave stage, parasitoid load, and the interaction term were included as fixed effects. The best models were chosen by AIC (lowest value) and included only shock stage. The effects of the predictor variable were determined by comparing models without shock stage to the best fit model using ANOVA with a chi‐squared test. A random intercept of individual was included in all models.

Parasitoid mass at eclosion was analyzed using linear mixed effects models with the “lme” function in the nlme package in R (v. 4.0.2). Individual wasp weight (total mass of wasps by sex/number of wasps by sex) was used as the response variable, and shock stage, parasitoid load, and sex were included as fixed effects, as well as all interaction terms. Individual host ID was included as a random intercept.

### 2.7 Temperature and duration experiment

Differences in parasitoid load (number of larvae found in hemocoel +number emerged) and the number of larvae that survived to emergence (temperature/duration experiment) were analyzed using linear mixed effects models using the “lme” function in the package “nlme” in R (v 4.0.2). Due to the low parasitoid hatching success in many of the treatments, it was not possible to conduct generalized linear models to analyze survival. Data from the control treatments were compared to data from both heat wave treatments to determine the effect of experiencing a heat wave. A subset of data containing only data from the heat wave treatments were analyzed separately to determine the effect of DMT (40°C or 42°C) and the length of exposure. Load and number emerged were log transformed to achieve normalcy of errors and homogeneity of variance. Daily maximum temperature (factor), days in heat wave (numeric), and the interaction term were included as fixed effects. All models included a random intercept of individual host ID.

A subset of WOWE hosts from each heat wave temperature and exposure time treatment combination (*n* = 5–6) were dissected to find evidence of wasp larvae that survived hatching, or melanized plaques assumed to be encapsulated parasitoid eggs. The difference in mass between WOWE hosts with wasp larvae and without wasp larvae was analyzed using a two‐sided Student's *t* test.

## 3 RESULTS

### 3.1 Developmental timing experiment

**FIGURE 2:**
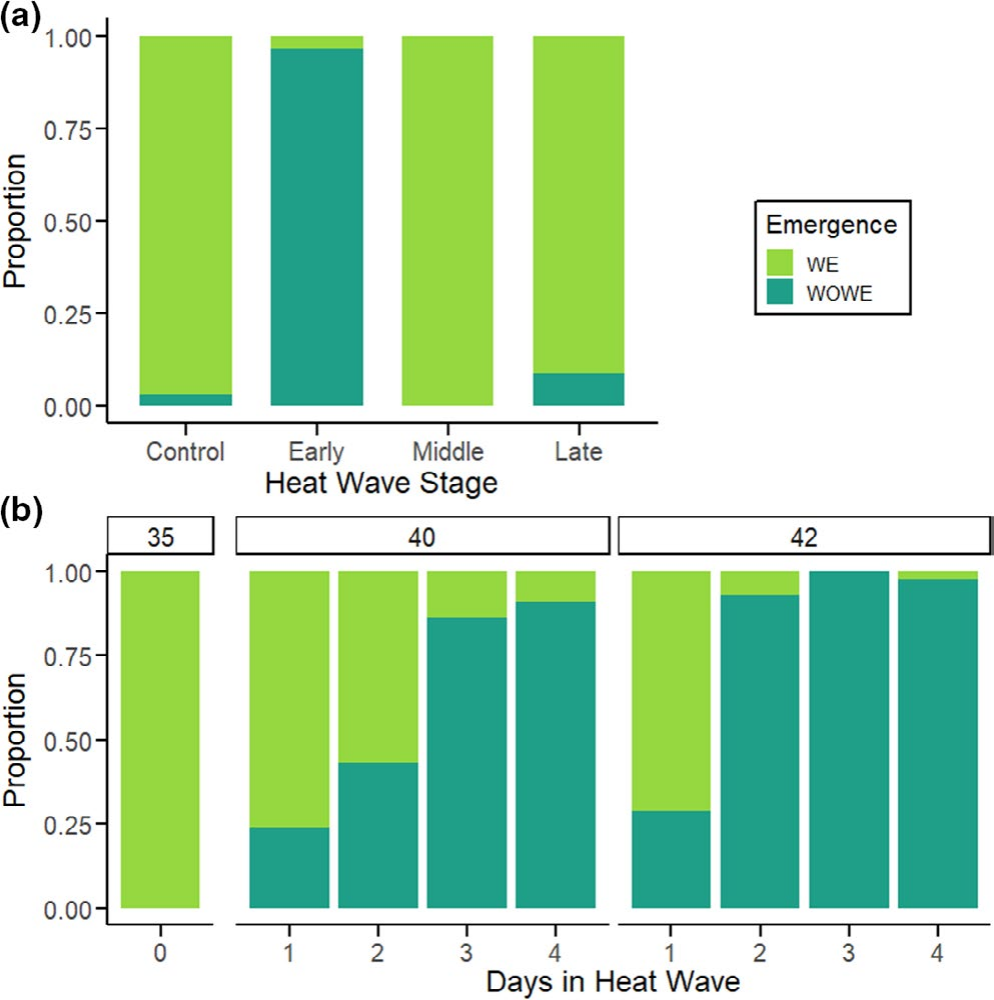
Outcomes of *M*. *sexta* hosts parasitized by *C*. *congregata* and subjected to heat wave (HW) treatment. Colors indicate the proportion of hosts in each treatment that had parasitoid emergence (green) or the WOWE phenotype (teal). a: Developmental timing experiment—Parasitized *M*. *sexta* were exposed to heat wave regime (DMT 42°C for 3 days) at various stages of parasitoid development. Early: same day as oviposition, parasitoids in the eggs stage. Middle: 5 days after oviposition, parasitoids in the 1st instar. Late: 2nd day of hosts’ 5th instar, parasitoids in the 2nd instar. b: Temperature/duration experiment—Parasitized *M*. *sexta* were exposed to one of two heat wave regimes (ramping, with 2 h at daily maximum temperatures [DMT]) of 40°C or 42°C for 1–4 days. Heat waves began on the same day as oviposition (1st day of the 3rd instar) Control individuals remained in the rearing temperature (DMT of 35°C)

**FIGURE 3:**
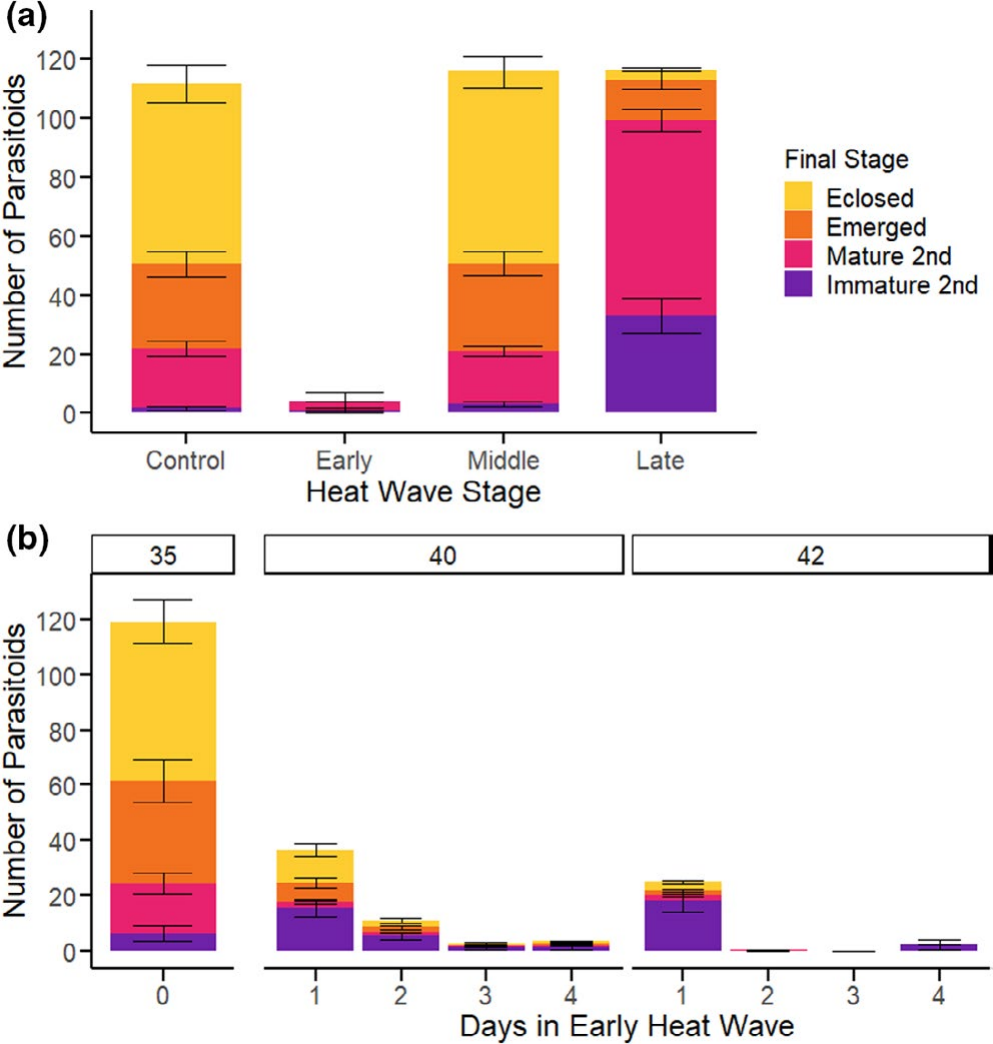
Parasitoid survival after heat shock is dependent on developmental stage (developmental timing experiment, a), and, early in development, on the daily maximum temperature (DMT) and the number of days exposed to the heat shock treatment (temperature/duration experiment, b). The mean number of parasitoids (hatched load: 2^nd^ instar and older) per host for each treatment is represented by bar height. Color indicates the mean number of parasitoids that reached each developmental stage: purple =immature 2^nd^ instars (within host hemocoel, anal vesicles extruded), pink = mature 2nd instars (within host hemocoel, anal vesicles retracted), orange = emerged from host (outside host, 3rd instar larvae and pupae), yellow = eclosed adults (successfully finished development). a: Parasitized caterpillars were exposed to a 3‐day heat wave (DMT of 2 h at 42°C) at 3 developmental time points in parasitoid development. Parasitized control caterpillars remained at the rearing treatment (25 ± 10°C, DMT = 35°C) throughout development. b: Parasitized caterpillar were exposed to heat wave treatments on the same day as parasitoid oviposition and were subjected to heat waves with one of two DMTs (40°C or 42°C), for a range of 1–4 days. Parasitized control caterpillars remained at the rearing treatment (25 ± 10°C, DMT = 35°C) throughout development. Error bars = *SE*

The presence or absence (WOWE) of wasp emergence from a host depended on the timing of heat wave (Figure 2a). Early heat wave (while the parasitoids are still eggs) nearly always resulted in WOWE hosts (Figure 2a). In contrast, heat waves later in parasitoid development never (Middle) or rarely (Late) produced WOWE hosts. Developmental timing of heat wave altered the size of hatched parasitoid load (the total number of unemerged and emerged wasp larvae: see Methods), and the distribution of the final developmental stages reached by the parasitoid (Figure 3a). Early heat waves presumably killed wasp embryos prior to hatching, resulting in very small loads and no emergence. Middle heat wave (during the parasitoid's 1st larval instar) did not disrupt wasp development, and this treatment had load numbers, parasitoid developmental distribution and survival to eclosion comparable to the control group (Figure 3a; Table 1). The survival of parasitized hosts in the Middle heat wave was much lower than controls, however (Table S1). Late heat wave (during the parasitoid's 2nd larval instar) caused significant mortality, reducing emergence (but not load) by 90% (binomial GLMM, *F*‐value = 202.36, *p*‐value < .0001; Figure 3, Table 1). Hosts in the Late treatment were found to have numerous parasitoid larvae upon dissection, unlike the majority of WOWE hosts. These parasitoid larvae ranged in developmental stage from early 2nd instars (small, anal vesicle still extruded) to mature 2nd instars that appeared, developmentally, able to emerge (large, anal vesicle retracted, some in the process of molting to 3rd (final) instar) (Figure 3a) (Fulton, 1940).

**TABLE 1:** Developmental timing experiment

Model	df	Ln(likelihood)	Δdf	*Χ* ^2^	*p*‐Value
A	Survival to emergence				
HW stage model	4	−515.75	–	–	–
Null model	2	−610.73	2	189.96	**<.0001**
B	Survival to eclosion				
HW stage model	4	−516.28	–	–	–
Null model	2	−607.76	2	182.96	**<.0001**

Notes

Survival of *C*. *congregata* to emergence (A) and eclosion (B) analyzed using generalized linear mixed effects models with binomial distributions. A random intercept of individual was included in each model. Models of best fit (with heat wave [HW] stage as the only fixed effect) were compared to null models using chi‐squared tests.

Bold values are significant, with *p* < .05.

**FIGURE 4:**
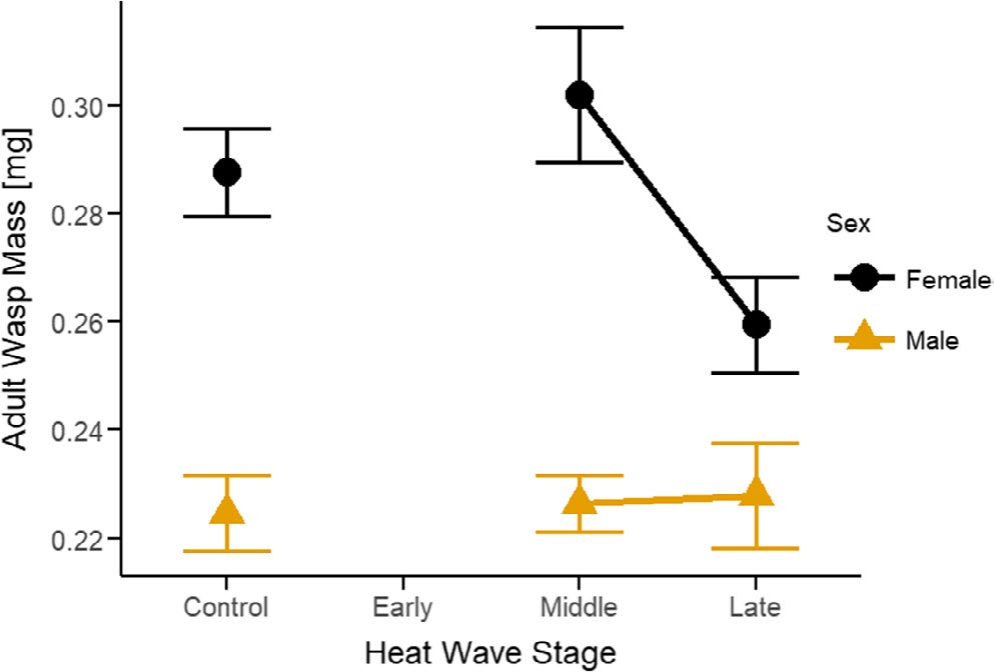
Developmental timing experiment—The effects of the developmental timing of heat waves on the adult mass of *C*. *congregata* (Females = black, ●; Males = yellow, ▲). Early heat shocks resulted in complete wasp mortality, and hosts displaying the WOWE phenotype. Mean adult parasitoid mass was strongly influenced by sex, with females being much larger than males. Male mass did not differ with developmental stage at heat shock. Females significantly increased in mass in the Middle treatment compared to controls. Females in the Late heat shock had reduced mass; this difference was not significant compared to controls, but the sample size in the Late treatment was very low compared to other treatments, due to increased mortality. Adult mass was determined by weighing all parasitoids per host en masse (separated by sex) and dividing by the number of parasitoids (by sex). Error bars = *SE*

Parasitoid adult mass was affected by heat waves at sensitive stages. Body mass of female and male adult wasps for the Middle heat wave was comparable to controls, but mean female mass was reduced in response to the Late heat wave (Figure 4). This difference was not statistically significant (potentially due to the small sample size of surviving adult wasps), but displayed a strong qualitative trend (LMM, *F*‐value = 1.766, *p*‐value = .1741; Figure 4). Adult parasitoid mass was significantly affected by load size, and the direction of effect depended on wasp sex and heat wave stage (LMM, *F*‐value = 9.432, *p*‐value = 0.0027; Table 2, Figure S1). As in other braconid wasps, the sex ratio for *C*. *congregata* was strongly male‐biased, but sex ratio of eclosing adults did not differ systematically among treatments.

**TABLE 2:** Developmental timing experiment: Linear mixed effects model of *C*. *congregata* adult mass

	Parasitoid adult mass		
	df	*F*	*p*‐Value
HW stage	2	1.776	.1741
Sex	1	138.08	**<.0001**
Load	1	9.43	.**0027**
HW stage: Sex	2	2.65	.0770
HW stage: Load	2	3.18	.**0456**
Sex: Load	1	2.10	.1512
HW stage: Sex: Load	2	2.99	.**0561**

Notes

Developmental heat wave (HW) stage, wasp sex, load, and interaction terms were included as fixed effects. A random intercept of individual was included in the model. Terms shown below were included in the model of best fit, selected using AIC.

Bold values are significant, with *p* < .05

### 3.2 Temperature and duration experiment

The developmental timing experiment used a heat wave of three days with a maximum diurnal temperature of 42 °C (see Methods). Given the dramatic effects of Early heat wave (Figures 2a and 3a), we determined how the maximum temperature and duration of early heat wave impacted wasp and host success. One day in the heat wave regimen early in development (1 exposure to DMT of 40°C or 42°C for 2 h) was sufficient to cause 25–30% hosts to fail to have wasp emergence (WOWE) (Figure 2b). Increasing the number of exposures increased the proportions of WOWE hosts; at 3–4 days in the heat wave regimen, 86–100% of hosts had no wasp emergence, especially at DMT of 42°C (Figure 2).

The total number of *C*. *congregata* larvae that hatched and developed within a host (number that emerged + the number found in hemocoel) was significantly lower in hosts that experienced a heat wave with a DMT of 40°C or 42°C, compared to the control group (LMM, *F*‐value = 19.2382, *p*‐value < .0001; Figure 3b; Table 3). Exposure to one day in heat wave (2 h at DMT) was sufficient to reduce mean parasitoid numbers by 3 to 5 fold (40 and 42°C, respectively), when compared to hosts at control temperatures. One day in the heat wave also changed the distribution of parasitoid final developmental stages: increasing the proportion that died as immature 2nd instar larvae (Figure 3b). Increased days in the heat wave regime continued to decrease parasitoid numbers significantly (LMM, *F*‐value = 4.209, *p*‐value = 0.0423), though the heat wave temperatures did not significantly differ (LMM, *F*‐value = 0.0275, *p*‐value = .8687). The majority of dissected hosts (with wasp emergence) had numerous melanized plaques found in the hemocoel, which we assume to be encapsulated parasitoid eggs. Hosts that were exposed to early heat waves also displayed significantly lower numbers of wasp larvae emerging (LMM, *F*‐value = 42.0592, *p*‐value < .0001), and this number decreased significantly with increasing number of days in the heat wave treatment (LMM, *F*‐value = 7.37697, *p*‐value = .0078; Figure 3b; Table 3). Significantly fewer parasitoids emerged from hosts exposed to DMT of 42°C than 40°C (LMM, *F*‐value = 6.64492, *p*‐value = .0114; Figure 3b; Table 3B).

**TABLE 3:** Temperature/duration experiment: Analysis of *C*. *congregata* hatched load and number emerged using linear mixed effects models to examine the effects of heat wave (HW) treatments

Treatment	df	*F*	*p*‐Value
A	Hatched load		
Control vs Heat wave	2	19.238	**<.0001**
Heat wave temp	1	0.0275	.8687
Days in heat wave	1	4.2090	.**0423**
HW temp*HW days	1	0.4476	.5047
B	Number emerged		
Control vs Heat wave	2	42.059	**<.0001**
Heat wave temp	1	6.645	.**0114**
Days in heat wave	1	7.377	.**0078**
HW temp * HW days	1	0.0273	.8691

Notes

Hatched load and number emerged are log transformed for normalcy. Shaded rows indicate analyses conducted on full data set, comparing control individuals (DMT = 35°C) against all HW groups (DMT = 40°C or 42°C). Unshaded rows are the results of analyses conducted on a subset of the data containing only HW individuals, with fixed effects of HW temperature (factor) and days in HW (numeric). All models included a random intercept of host ID. A: Parasitoid hatched load (number emerged + number found in hemocoel), or the number that survived to hatching. B: Number of parasitoids that survived to emerge from the host.

Bold values are significant, with *p*<.05

**FIGURE 5:**
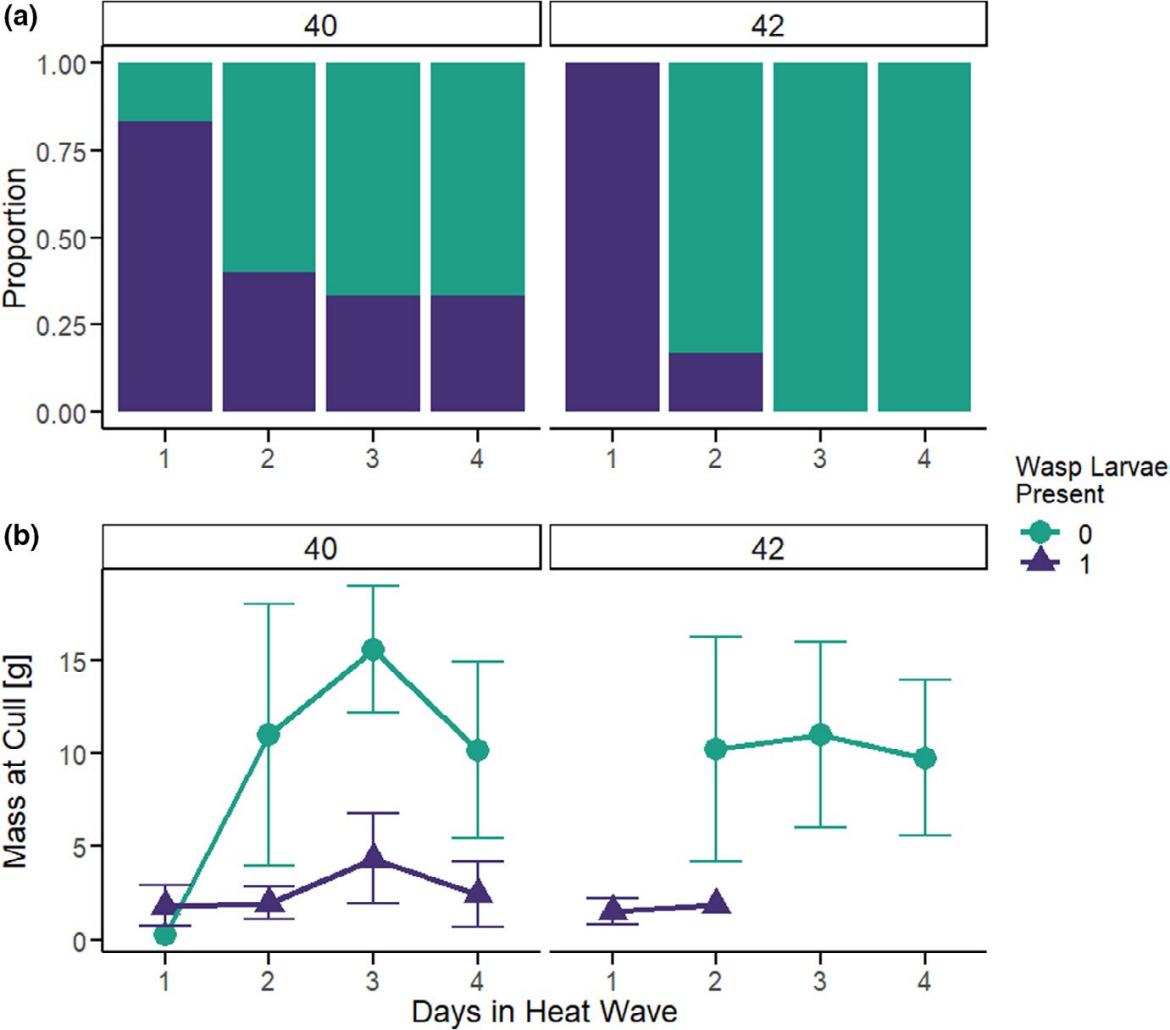
Dissected WOWE hosts differed in the presence/absence of parasitoid larvae found within the hemocoel. (a) The proportion of dissected WOWE hosts in which parasitoid larvae were found decreased as the number of days in the heat wave increased. (b). WOWE hosts with wasp larvae had significantly lower mass at culling than those without, though the variation around the mean is large for the latter group; error bars = *SD*. The distribution of mass for WOWE hosts differs dependent on how long they remained in the heat wave treatment; WOWE hosts in the 1 day treatment were consistently small, within the range of mass for parasitized hosts at wasp emergence. As exposure to the heat wave increased, mass at culling shifts to a bimodal distribution; some hosts remain small, while others attain masses up to 20 g

A subset of WOWE hosts were frozen for dissection from the temperature/duration experiment (5–6 per treatment), to determine whether parasitoid larvae were present within the hemocoel that never emerged. Some number of dissected WOWE hosts did have wasp larvae (usually 1^st^ or immature 2nd instars), and the proportion with wasp larvae decreased as the number of days in the heat wave treatment increased (Figure 5). The number of wasp larvae found within WOWE hosts was small, consistent with the low load numbers seen in hosts with wasp emergence (Figure 3b; Table 3A). These WOWE hosts with wasp larvae often had abundant melanized plaques. WOWE hosts with no wasp larvae lacked melanized plaques (though see Discussion) and were significantly larger than WOWE hosts with wasp larvae: up to 2–3× greater in mass (*t* test, df = 33.5, *p*‐value < .0001; Figure 5b). WOWE hosts that lacked wasp larvae had greater variance in mass than WOWE hosts with wasp larvae (mean mass = 10.728 ± 5.228 g and mean mass = 2.087 ± 1.325 g, respectively).

## 4 DISCUSSION

Variation in thermal tolerance among life stages is widespread in ectotherms (Kingsolver et al., 2011; Pandori & Sorte, 2019), but general patterns across ontogeny for insects have yet to emerge (Kingsolver & Buckley, 2020). Several recent studies with insects show that eggs have lower heat tolerance than larvae (Klockmann & Fischer, 2017; Klockmann et al., 2017; MacLean et al., 2016). Our results indicate a more complex pattern in *C*. *congregata*: the parasitoid is most sensitive to high temperatures both during early embryonic development and late in larval development prior to emergence and pupation. Interestingly, the parasitoid appears to be resilient to heat waves during the intermediate portion of its larval development; while host mortality was increased in our Middle heat wave treatment, the surviving hosts produced parasitoids with comparable survival, development time, and adult mass to controls that never experienced a heat wave. The *M*. *sexta* hosts themselves could be sensitive to high temperatures during this developmental stage (Kingsolver et al., 2021), but this remains to be fully explored in parasitized caterpillars.

Heat waves early in development could reduce *C*. *congregata* in two ways. First, high temperatures could disrupt embryonic development and reduce hatching success and hatchling survival. Our findings in the temperature/duration experiment provide support for this hypothesis, as lengthening the duration of the heat waves resulted in lower numbers of parasitoids (decreased hatching success) and abnormal morphology of parasitoid larvae (see below). Alternatively, high temperature stress could disrupt the action of the CcBV virus, which suppresses key elements of the host's immune system. Disrupting viral gene expression would enable the host immune system to encapsulate the parasitoid eggs and prevent them from hatching (Chevignon et al., 2015; Seehausen et al., 2017). High temperatures have been shown to upregulate host immune function as well, which could lead to parasitoid death if the viral immune suppression was unsuccessful (Seehausen et al., 2017). Further work is required to distinguish between these two hypotheses.

Heat waves late in parasitoid development (2nd larval instar) likely increase parasitoid mortality by different mechanisms. The majority of nutrient uptake and growth takes place during the parasitoid's 2nd instar, during which larvae increase 35‐fold in mass (Beckage & Riddiford, 1978, 1983). When parasitized *M*. *sexta* are starved during the parasitoid's 2nd instar, the majority of parasitoid larvae fail to emerge from the host's cuticle (Beckage & Riddiford, 1983; Bentz & Barbosa, 1992). Heat stress during this crucial period could disrupt nutrient uptake by the wasp, or nutrient release by the host, preventing *C*. *congregata* larvae from emerging and completing development. Our results suggest that parasitoids in the Late heat wave treatment had reduced adult female mass (Figure 4). Upon dissection, hosts in the Late treatment had a large number of immature 2nd instar parasitoid larvae (anal vesicles still extruded), indicating that development within a host had become asynchronous (Figure 3). Alternatively, the synthesis and release of ecdysteroid hormones necessary for parasitoid emergence could have been disrupted by the exposure to high temperature stress (Gelman et al., 1999).

A previous study in this system showed that high, diurnally fluctuating temperatures (e.g., 30 ± 10°C) throughout development eliminates wasp emergence, while having no lethal effect on unparasitized *M*. *sexta* (Moore et al., 2021a, 2021b). The current study demonstrates that even a single exposure to a DMT of 40°C or 42°C during early parasitoid development significantly reduced hatching success and survival to emergence; 3–4 day‐long heat waves at either DMT result in complete failure of wasp emergence (Figures 2 and 3). This finding is consistent with recent studies in other insects that document how single, high‐temperature events or heat waves can reduce growth, survival, and reproduction (Ma et al., 2015, 2018; Zhang, Chang, et al., 2015; Zhang, Rudolf, et al., 2015; Zhao et al., 2019). Strikingly, the fitness consequences of a high‐temperature event or heat wave for *C*. *congregata* depend critically on the timing of the event during the parasitoid's life cycle. As a result, predicting the consequences of extreme temperature events in nature will be even more challenging in this system (Bailey & van de Pol, 2016; Chevin & Hoffmann, 2017; Grant et al., 2017; Harris et al., 2018; Stoks et al., 2017). Whether this is the case for other parasitoids is unknown.

As reported in several other host–parasitoid systems (Furlong & Zalucki, 2017), the heat tolerance of *M*. *sexta* is considerably greater than that of *C*. *congregata*. For example, a single, 3‐day heat wave (DMT 42°C, at similar time points used in our study) during larval development has minimal effects on survival or final size in unparasitized *M*. *sexta* larvae, but causes complete parasitoid mortality early in the development of *C*. *congregata* (Casey, 1976; Kingsolver et al., 2021). Disruption of the parasitoid's early development does not rescue the host (all die prior to pupation), and these WOWE hosts exhibit a range of abnormal phenotypes, including greatly extended larval life spans and unusually high body masses which, to our knowledge, have never been observed in unparasitized *M*. *sexta*, but has been simulated by injection of CcBV into unparasitized caterpillars (Figure 5) (Dushay & Beckage, 1993; Moore et al., 2021a, 2021b). Successful development of *C*. *congregata* relies on a combination of an endogenous polydnavirus (suppresses host immune system and disrupts host hormonal regulation), and the action of the parasitoid larvae themselves (cease host feeding and locomotion before emergence) (Adamo et al., 2016; Beckage & Riddiford, 1982; Beckage et al., 1994). High temperatures that kill wasp larvae early in development (either directly or through disruption of viral action to suppress the host immune system) could cause the effects we see in hosts WOWE: the absence of living *C*. *congregata* larvae results in continual feeding by the host, and CcBV transcripts prevent pupation, creating long‐lived, massive *M*. *sexta* caterpillars. This hypothesis is supported by our preliminary findings that the WOWE hosts that grew abnormally large (>10 g) completely lacked parasitoid larvae in their hemocoel; WOWE hosts that had any wasp larvae survive to hatching remained at masses within the expected range at parasitoid emergence (1–5 g) (Figure 5).

The differing thermal tolerances of insect hosts and parasitoids may have important consequences for the effects of climate change and extreme temperature events on host–parasitoid interactions (Furlong & Zalucki, 2017). Each ecological player has a window of thermal vulnerability dictated by their intrinsic responses to temperature stress, and these windows shift depending on developmental stage and the nature of the thermal stress. When these windows of vulnerability or sensitivity do not coincide between interacting species (e.g., parasitoids dying at lower temperatures than their hosts), then ecological systems are at risk of breaking down. This can be seen more generally in the effects of climate change on the interactions between species involved in symbiotic relationships, such as coral and their endosymbionts, and amphibian hosts and chytrid fungal pathogens (Bradley et al., 2019; Schoepf et al., 2015). Our studies demonstrate that extreme temperature events can disrupt the interactions among host, parasitoid, and possibly endosymbiont—potentially leading to the death of all three players—but the outcome depends critically on the timing of the event within the parasitoid's life cycle. Predicting the effects of extreme temperature events in such interacting ecological systems represents a major challenge for ecologists; our study illustrates the importance of studying thermal responses of interacting species across their life cycles.

## CONFLICT OF INTERESTS

The authors have no competing interests to report.

## AUTHOR CONTRIBUTION

**Megan Elizabeth Moore:** Conceptualization (lead); Data curation (lead); Formal analysis (lead); Investigation (lead); Methodology (lead); Visualization (lead); Writing – original draft (lead). **Christina A. Hill:** Data curation (supporting); Investigation (supporting); Methodology (supporting); Project administration (supporting); Writing – review & editing (supporting). **Joel G. Kingsolver:** Conceptualization (supporting); Formal analysis (supporting); Funding acquisition (lead); Project administration (supporting); Resources (lead); Supervision (lead); Validation (equal); Visualization (supporting); Writing – original draft (supporting); Writing – review & editing (lead).

## Supporting information

Supplementary Material

## Data Availability

Data and statistical analyses available from the Dryad Digital Repository: https://doi.org/10.5061/dryad.8kprr4xn4 (Moore et al., 2021).
