# Supplementary material for: Developmental timing of extreme temperature events (heat waves) disrupts host–parasitoid interactions
Source: Ecol Evol. 2022 Mar 18;12(3):e8618. doi: 10.1002/ece3.8618 (PMC8932226; doi:10.1002/ece3.8618)
Supplement: Supplementary file 1 — Supplementary Material [file ECE3-12-e8618-s001.docx]

Table S1. The number of parasitized *M. sexta* in the developmental timing experiment (**A**) and the temperature/duration experiment (**B**) that died, wandered, or were included in final figures and analyses (final sample size).

**A**

| **Heat Wave stage** | **Died** | **Wandered** | **Final sample size** |
| --- | --- | --- | --- |
| Control | 3 | 1 | 36 |
| Early | 7 | 4 | 31 |
| Middle | 34 | 4 | 59 |
| Late | 19 | 2 | 46 |

**B**

| **DMT** | **Duration** | **Died** | **Wandered** | **Final sample size** |
| --- | --- | --- | --- | --- |
| 35 | 0 | 5 | 3 | 21 |
| 40 | 1 | 7 | 4 | 46 |
|  | 2 | 3 | 5 | 44 |
|  | 3 | 8 | 6 | 44 |
|  | 4 | 12 | 10 | 44 |
| 42 | 1 | 10 | 2 | 45 |
|  | 2 | 16 | 6 | 42 |
|  | 3 | 4 | 3 | 46 |
|  | 4 | 8 | 7 | 46 |


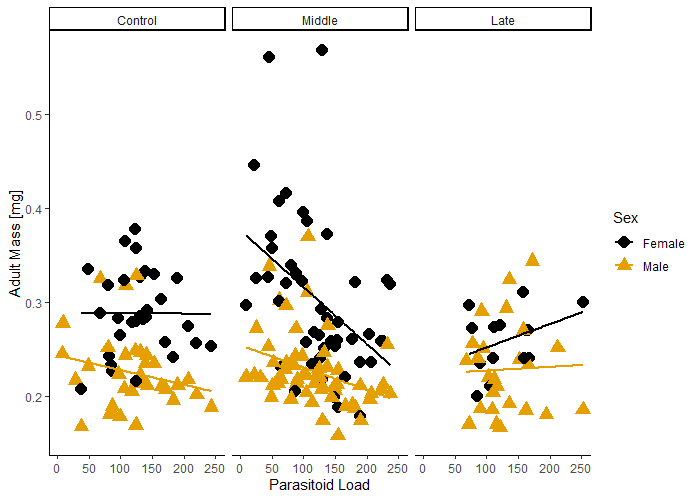


Fig S1. Adult parasitoid mass differs by sex (female = black, ●; male = yellow, ▲) and load, dependent on the stage exposed to the heat wave treatment. Adult mass was measured by weighing all parasitoids for a host, separated by sex, then dividing by the number of wasps weighed.


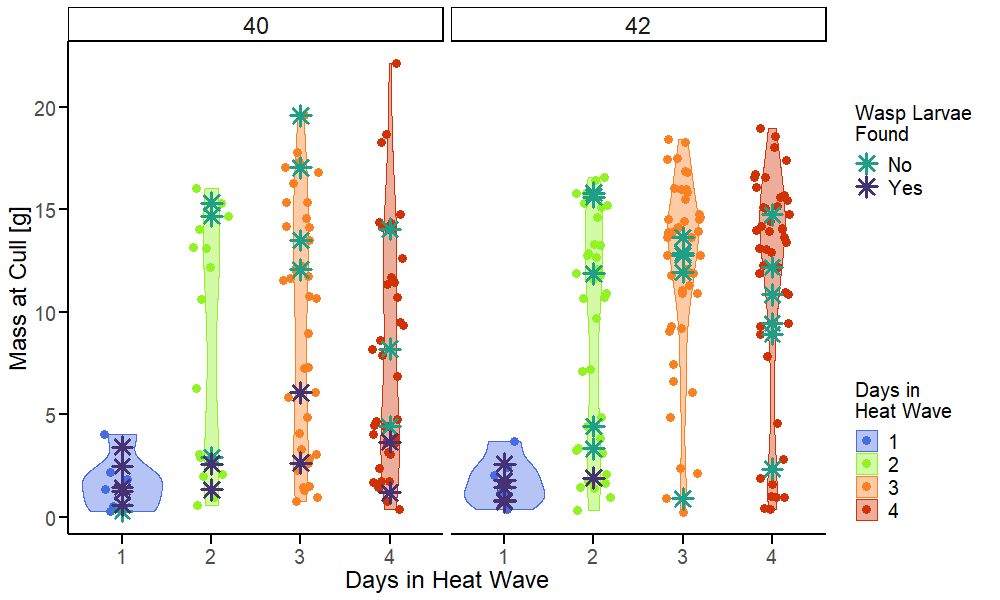


Fig S2. Dissected WOWE hosts differed in the presence/absence of parasitoid larvae found within the hemocoel. The proportion of dissected WOWE hosts in which parasitoid larvae were found decreased as the number of days in the heat wave increased. All WOWE hosts were culled 2 weeks after the molt to 5^th^ if no wasp emergence was present. The distribution of mass for WOWE hosts differs dependent on how long they remained in the heat wave treatment; WOWE hosts in the 1 day treatment were consistently small, within the range of mass for parasitized hosts at wasp emergence. As exposure to the heat wave increased, mass at culling shifts to a bimodal distribution; some hosts remain small, while others attain masses up to 20g. The higher DMT treatment (42°C) appears to have qualitative effects on the distribution of mass in WOWE hosts; the bimodality of mass distribution is strengthened in days 3 and 4 at 42°C compared to 40°C, and the presence of wasp larvae in dissected WOWE hosts is 0. However, this trend requires further investigation to be supported. ● indicate undissected WOWE hosts, while * indicate hosts that were dissected to determine presence of wasp larvae.
